# A study of Drynaria fortunei in modulation of BMP–2 signalling by bone tissue engineering

**DOI:** 10.3906/sag-2001-148

**Published:** 2020-08-26

**Authors:** Guo-Chung DONG, Tzn-Yuan MA, Chi-Han LI, Chih-Ying CHI, Chao-Ming SU, Chih-Ling HUANG, Yan-Hsiung WANG, Tzer-Ming LEE

**Affiliations:** 1 Institute of Biomedical Engineering and Nanomedicine, National Health Research Institutes, Miaoli County Taiwan; 2 Center for Fundamental Science, Kaohsiung Medical University Kaohsiung Taiwan; 3 School of Dentistry, College of Dental Medicine, Kaohsiung Medical University, Kaohsiung Taiwan

**Keywords:** BMPR-1A, naringin, bone tissue engineering

## Abstract

**Background/aim:**

*Drynaria fortunei* (Gusuibu; GSB) is a popular traditional Chinese medicine used for bone repair. An increasing number of studies have reported that GSB induces osteogenic differentiation in bone marrow mesenchymal stem cells (BMSCs). These results provide insight into the application of GSB for bone tissue engineering techniques used to repair large bone defects. However, few studies have described the molecular mechanisms of GSB.

**Materials and methods:**

In the present study, the effects of GSB and naringin, a marker compound, on the binding of BMP-2 to BMPR and BMP-2-derived signal transduction were investigated using surface plasmon resonance (SPR) and coculturing with BMPR-expressed cell line, C_2_C_12_, respectively. Furthermore, naringin was also used to prepare naringin contained scaffolds for bone tissue engineering. The physical and chemical properties of these scaffolds were analysed using scanning electron microscopy (SEM) and highperformance liquid chromatography (HPLC). These scaffolds were cocultured with rabbit BMSCs in vitro and implanted into rabbit calvarial defects for bone repair assessment.

**Results:**

The results showed that GSB and naringin affect the binding of BMP and BMPR in SPR experiments. GSB is a subtle BMP modulator that simultaneously inhibits the binding of BMP-2 to BMPR-1A and enhances its binding to BMPR-1B. In contrast, naringin inhibited BMP-2 binding to BMPR-1A. In vitro studies involving the phosphorylation of signals downstream of BMPR and Smad showed that GSB and naringin affected stem cell differentiation by inhibiting BMPR-1A signalling. When using GSB for bone tissue engineering, naringin exhibited a higher capacity for slow and gradual release from the scaffold, which promotes bone formation via osteoinduction. Moreover, control and naringin scaffolds were implanted into rabbit calvarial defects for 4 weeks, and naringin enhanced bone regeneration in vivo significantly.

**Conclusion:**

GSB and its marker compound (naringin) could inhibit the binding of BMP-2 and BMPR-1A to control cell differentiation by blocked BMPR-1A signalling and enhanced BMPR-1B signalling. GSB and naringin could be good natural BMP regulators for bone tissue engineering.

## 1. Introduction

Many types of Chinese herbal medicines have been shown to be effective for bone regeneration[1–4]. Some of Chinese herbal medicines had provided the effect on bone healing, such as *Carthami flos* [5], *Epimedii* [6], and *Drynaria fortunei* (Gusuibu; GSB) [7, 8] et al. GSB is a popular traditional Chinese medicine (TCM) used for bone repair [9, 10], but its mechanism of action remains unclear. GSB has been demonstrated as an effective herb to stimulate the proliferation of human ﬁbroblasts [11], foetal rat periosteal osteoprogenitor cells [12], foetal rat calvaria osteoblasts [13], and human osteoblasts [14]. Naringin is the main active ingredient of total flavonoids of *Rhizoma drynariae* , which inhibit retinoic acid-induced osteoporosis in rats [15], increase bone morphogenetic protein (BMP)-2 expression, induce bone formation [16–18], and enhance the proliferation and osteogenic differentiation of human BMSCs in osteoporosis [19–21].

The routes of administration of Chinese herbal medicines also are oral, injection, and external [22]. As tissue engineering progresses, Chinese herbal medicines had combined with scaffold as a novel therapeutic administration [23–25]. Preliminary studies have shown that implanted biomaterials containing GSB can enhance bone repair through osteoconduction [12,26,27]. These results provide insight into the application of GSB for bone tissue engineering to repair large bone defects. The current literature has deeply explored the traditional dosage mechanism of Chinese herbal medicine, the effect of implant treatment methods is still mostly evaluated the effective, but the treatment mechanism and effect are still unclear.

An increasing number of studies have reported that GSB can induce osteogenic differentiation in mesenchymal stem cells (BMSCs) [28]. GSB and its marker compound naringin had confirm the effect on osteogenesis, and which had proved enhanced osteogenesis and the naringin-mediated upregulation of ALP activity [21,29]. Even the pathway of naringin enhanced ALP activity had been proven, the mechanism by which naringin binds to receptor has not been elucidated. BMP is important for osteogenic differentiation. Binding with BMP receptors (BMPR-1A and BMPR-1B) initiates BMPR signalling, resulting in cell proliferation and differentiation [30,31].

In the study, the major rationale are that Chinese herbal medicines can regulate the binding of protein to receptor [32–34]. Our hypothesis is some compounds of GSB regulate BMPR function to enhance osteogenesis. For this study, our aim is investigated the effects of GSB on the binding of BMP-2 and BMPR. Furthermore, naringin was also used in binding assays in scaffolds incorporating BMP-2 to examine signal transduction and its role in bone tissue engineering. 

## 2. Materials and methods

### 2.1. Chemicals and reagents

The CM5 sensor chip, HBS-EP buffer (10 mM HEPES, 0.15 M NaCl, 3.4 mM EDTA, and 0.05% surfactant P20, pH 7.4) and the amine-coupling kit containing N-hydroxysuccinimide (NHS), N-ethyl-N-(3-diethylaminopropyl) carbodiimide (EDS) and ethanolamine hydrochloride were obtained from Biocore AB (Uppsala, Sweden). Naringin, phosphate-buffered saline (PBS) and sodium anhydrate were obtained from Sigma-Aldrich Chemie GmbH (Taufkirchen, Germany). Human BMP-2 and human BMPR-1A were purchased from R&D Systems Inc. (Minneapolis, MN, USA). Recombinant human BMPR-1A was constructed as a disulfide-linked homodimeric protein comprising the extracellular domain (645 aa) of human BMPR-1A fused to the Fc region of human IgG via a linker peptide. 

### 2.2. Preparation of Drynaria fortunei extractions

*Drynaria fortunei* (Gusuibu; GSB) was purchased from the Scientific Traditional Chinese Medicine Company in Taiwan. Twenty grams of GSB was extracted with methanol (200 mL) by stirring at room temperature for 20 min. The methanol extracts were collected and filtered through filter paper (No.1, code no. PW300-1125, pore size = 10 µm, TOYO Inc., Tokyo, Japan). Extraction was repeated 3 times. All filtrates were collected and dried under reduced pressure. Next, 10 mL of PBS was added to dissolve the extract. Subsequently, the solution was centrifuged at 8000 rpm for 60 min. The supernatant was collected and completely dried under reduced pressure. 

### 2.3. SPR analysis

A BIAcore 2000 (Biacore, Uppsala, Sweden) was employed for real-time biospecific interaction analysis. The binding analysis was performed at 25 °C with a flow rate of 10 mL/min. In general, BMPR proteins were immobilized on a layer of carboxylated dextran on a CM5 sensor chip (research grade; Biacore) through amine coupling. The independent variables (naringin or GSB) were diluted in PBS running buffer to a final concentration prior to injection. The binding of GSB or naringin was recorded in real time, resulting in a sensorgram of relative signal changes (RU). The dependent variable was the binding constant of BMPR-1A and BMP-2. The binding constant for immobilized BMPR-1A and BMP-2 was determined using BIA evaluation 3.1 software (Biacore). GSB and naringin treatments used to examine BMP and BMPR binding were evaluated using SPR. Subsequently, the cells were washed with 50 mM NaOH buffer to remove bound material. 

**BMP-2** **signal transduction**

Mouse C_2_C_12_ muscle myoblasts overexpressing the BMPR were purchased from BCRC (60083, Taiwan). The cells were cultured in Dulbecco’s Modified Eagle’s Medium (DMEM, Biowest) containing 10% foetal bovine serum (FBS). For cell BMP-2 signal transduction, the subculture medium was changed to DMEM containing 2% horse serum (HS) (differentiation medium), and the cell density was 1 × 10^5^ cells/cm^2^. A concentration of 10 nM BMP-2 (R&D) was used to stimulate BMPR signal transduction. The medium was refreshed every three days during the BMP-2 stimulation period. To examine BMP-2 signal regulation (dependent variable), the independent variables of GSB or naringin were added to the culture medium in the test group, and dexamethasone, dorsomorphin and noggin were added to the control group. Subsequently, the cells were analysed using Western blotting. 

Total protein was extracted from cells plated onto a 6-cm Petri dish at a density of 5 × 10^4^ cells/cm^2^, and the protein concentration was measured using a BCA protein assay (Bio-Rad). The protein extracts were separated using 15% SDS-PAGE and subsequently transferred to PVDF membranes. The membranes were incubated with 5% skim milk for 1 h at RT to block nonspecific binding. For osteogenesis analysis, Smad and P38 phosphorylation and Smad and actin expression were detected using a primary antibody (1:1,000–1:20,000 dilution). After washing the membrane, a secondary antibody (1:5,000 dilution) was used, and the detected proteins were visualized using the ECL Plus Western blotting detection system (Cytiva GE Healthcare Life Sciences, Marlborough, MA, USA).

**Preparation of naringin scaffolds**

The GHG composites were prepared as previously described [12,19]. Briefly, a homogeneous 5 wt% gelatin solution was obtained by dissolving porcine gelatin (Bloom number 300, Sigma Chemical Co., Saint Louis, MO, USA) in deionized water in a water bath at 40 °C. After the solution was stirred for 2 min, 0.6 g NaCl and hydroxyapatite ceramic particles (21223-1, Sigma) were mixed with the gelatin solution. The weight ratio of gelatin to hydroxyapatite was 1:3. Following vigorous stirring at 70 rpm for 20 min, the mixture became increasingly viscous and was subsequently transferred to 5 mL plastic syringes and frozen at –80°C for 24 h. After thawing at room temperature, the solidified mixtures were cut using a scalpel and shaped into cylindrical specimens of a particular size. The dried cylindrical composites had a diameter of 12 mm and a thickness of 2 mm. The porous samples were incubated in 1.0 wt% glutaraldehyde solution (G6257, Sigma) on ice to induce a cross-linking reaction, followed by rotation at 10 rpm at 4 °C for 24 h away from light. Subsequently, the samples were incubated in 0.2 M glycine solution for 2 h to remove the residual cross-linking reagent. The samples were then washed 3 times with deionized water and incubated at 4 °C for 24 h. Subsequently, the GHG samples were washed 3 times with deionized water and stored at –80 °C for 24 h. Finally, the GHG samples were freeze-dried for 4 days and stored in dry box prior to use.

The *Nar* scaffolds were prepared using a procedure similar to that used to prepare the GHG composites. A homogeneous 5 wt% gelatin solution was obtained by dissolving porcine gelatin in naringin solutions of varying concentrations ranging from 0to 100 mg/mL; the optimal concentration was determined from the results of the release experiments. All samples were sterilized via UV exposure prior to use.

The cross-sectional morphology of the scaffolds was examined under a Hitachi S-3000N (Hitachi Microscopes, Tokyo, Japan) scanning electron microscope. The test sample was frozen and dried using the previously described procedure. The dried sample was immediately sputter coated with gold for further SEM observation. The average pore size in the cross-section was evaluated based on measurements of the pores in SEM micrographs. 

**Release assay of naringin scaffolds**

In the present study, the independent variables of *Nar* scaffolds, containing 0, 10, or 100 mg/mL of naringin, were used as test samples to evaluate the naringin release capability (dependent variable). To obtain consistent results, we repeated the release assay 5 times.

The examination of naringin release from the scaffolds was conducted using an incubation experiment and high-performance liquid chromatography (HPLC) analysis of the solution. Briefly, cylindrical scaffolds were weighed and incubated in glass sample bottles containing approximately 25 mL of DMEM solution. The volume to weight ratio was 50 mL/g. All samples were placed in a water bath and agitated. The temperature was set at 37 °C. The experiment was conducted for 14 days. On days 1, 2, 3, 5, 7, 14, and 100 µL, samples of the incubated solution were collected and filtered for subsequent HPLC analysis.

All filtered solutions were analysed using HPLC to determine the amount of naringin released from the scaffolds. The samples were filtered and analysed using an Agilent Technologies 1200 Series HPLC instrument equipped with a Quat Pump (G1311A) and a variable wavelength detector (G1314B) at 283 nm with an optical unit upgrade and computer software (ChemStation for LC 3D Systems rev.B.04.01sp1). Separation was conducted on a Discovery BIO Wide Pore C18 column (4.6 × 250 mm, 5 µm) from Supelco Inc. (Bellefonte, PA, USA). The samples and standards were injected using a Rheodyne 100-ml loop. The mobile phase containing a mixture of ACN and water was passed through a 0.22-mm PVDF filter. The gradient was maintained at 10%:90%, 10%~30% for 3 min. The flow rate was 1 mL/min. The obtained data were analysed using a ChemStation for LC 3D Systems rev.B.04.01sp1.

**Culture of BMSCs with the naringin scaffold**

BMSCs were aspirated from the iliac crests of mature male New Zealand white rabbits weighing 2.5–3.0 kg (purchased from the Livestock Research Institute, Council of Agriculture, Executive Yuan, Tainan, Taiwan) under total anaesthesia. All procedures were performed according to the National Institutes of Health Guidelines for the Use of Laboratory Animals and approved by the Institutional Animal Care and Use Committee of National Health Research Institutes. The rabbits were intramuscularly anaesthetised with Zoletil 50 (Virbac Co., Taipei, Taiwan) and 2% Rompun solution (Bayer AG, Leverkusen, Germany) (1:1 ratio, 0.2 mL/kg) in an aseptic animal operation room. The aspiration syringe was rinsed with sodium heparin (5000 U/mL; Chunghwa Chemical & Pharmaceutical Co., Taipei, Taiwan) to prevent clotting. Approximately 5 mL of bone marrow aspirates was harvested and added to low-glucose DMEM (L-DMEM; Gibco, Grand Island, NY, USA) supplemented with 10% FBS (Gibco), 1% penicillin/streptomycin (Gibco) and 3.7 g/L sodium bicarbonate. The cells were plated in a 75cm2 cell culture flask (Costar, Cambridge, MA, USA) and incubated at 37 °C under 5% CO_2_. The medium was changed after 24 h to remove nonadherent cells, and the adherent cells were reincubated. When the cells cultured in the flasks were almost confluent, they were detached using 0.25% trypsin/EDTA (Sigma) for 5 min at 37 °C. After primary culture, the cells were subcultured at 37 °C under 5% CO_2_. The culture medium was refreshed every 2 days. Cells in the second or third passage were used in the following experiments. 

A dynamic culture system was used to improve the exchange of nutrients and waste products between the interior and exterior of the scaffold. In addition, the culture system provided a mechanical stimulus to the cells. A total of 1 × 10^6^ BMSCs were loaded onto the sterilised scaffold and incubated to enable infiltration. After 5 mL of culture medium was added, the cell-seeded scaffold was cultured at 37 °C in a 5% CO_2_ atmosphere for 1 day. The seeded scaffold was subsequently placed in a spinner flask containing 250 mL of differentiation medium, with magnetic stirring at 70 rpm for 10 h, followed by stirring at 50 rpm for 2 weeks. The differentiation medium comprised high-glucose DMEM supplemented with 10% FBS, 1% penicillin/streptomycin, 3.7 of g/L sodium bicarbonate, 0.11 of g/L sodium pyruvate, 50 g/mL of L-ascorbic acid (Sigma), 10 mM b-glycerophosphate (Sigma), and 10–8 M dexamethasone (Sigma). The apparatus was placed in a CO_2_ incubator. The medium was replaced every 3 days.

After culturing with the scaffolds, the cross-sectional morphology of BMSCs was observed under a TM-1000 SEM (Hitachi). The test sample was frozen and dried following the aforementioned procedure. The dried sample was immediately sputter coated with gold for further SEM observation. 

**Animal model of large bone defect reconstruction**

In the present study, we used the independent variable ( *Nar* scaffold) to verifying bone repair (dependent variable) by in vivo study. Experimental cranial implantation was conducted on 20 adult male New Zealand white rabbits. All animals were anaesthetised with intramuscular injections of a combination of Zoletil 50 and 2% Rompun solution. The head of each rabbit was shaved and sterilized with 10% povidone-iodine solution (Chou Jen Pharmaceutical Co., Nantou, Taiwan). The cranial surface was exposed through a midline incision, and the overlying parietal periosteum was subsequently excised. A full-thickness circular defect of the parietal bone with a diameter of 12 mm was created using a drilling burr. The calvarial bone defects were filled with the sterile naringin and control scaffolds to evaluate their osteogenerative characteristics. Anesthetized animals were postoperatively sacrificed by administering an overdose of sodium pentobarbital (100 mg/kg) at 8 weeks. The craniectomy sites involved the removal of 2–3 mm of contiguous bone from each skull.

The bone defect repair was radiographically and histologically evaluated. The specimens were fixed using 10% phosphate-buffered formalin solution for 48 h and subsequently radiographed using X-O CT (70 keV, FOV: 3.7 cm, resolution: 65 μm, PET/SPECT/CT; tri-modality imaging system by Animal Molecular Imaging Core Facility (AMICF) in the Institute of Biomedical Engineering and Nanomedicine (IBEN) of the NHRI. The radiographic appearance of a calcified mass revealed new bone. The regenerated bone was quantified using a semiautomatic histomorphometric method. A satisfactory contrast was achieved between the implanted materials and the new bone tissue after setting consistent gray level sensitivity standards across all treatments using an image analyser system (Image-Pro Lite, Media Cybernetics, Silver Spring, MD, USA). The image analyser system, coupled to the microscope, was equipped with a phonic drawing tube, through which the image of the digitizing plate was projected over the optical field. The amount of new bone tissue was calculated by moving a cursor (calculated at the location of the cursor) on the digitizing plate, which produced a visible projection over the histologic field, expressed as a percentage of the ingrown bone tissue in the created bone defect.

For the histological analysis, the sample was fixed in 10 wt% neutral-buffered formalin solution (Sigma-Aldrich 252549, Taiwan) for 48 h, washed in PBS, and dehydrated in a graded series of ethanol solutions. Longitudinal sections of decalcified bone and implant (each 5 μm thick) were prepared and stained with hematoxylin and eosin (H&E) at a pathology core lab in the NHRI. The sections were observed under an optical microscope (Nikon ECLIPSE Ti-S).

### 2.4. Statistical analysis

All quantitative data are presented as means and standard derivations. The statistical analysis was performed using 1–way ANOVA, followed by a post hoc Fisher’s least significant difference test for multiple comparisons. Differences were considered significant at P < 0.05. Prior to each statistical test, a normal data distribution was verified using normal probability plots.

## 3. Results

### 3.3. BMP-2 signalling

**Figure 1:**
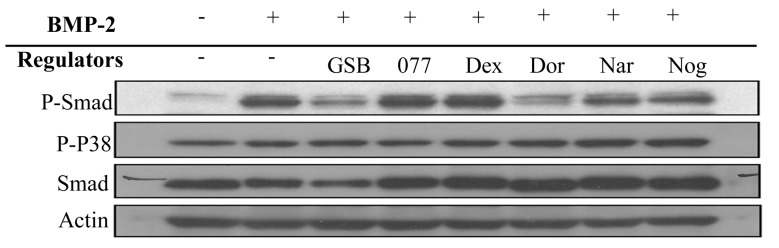
Phosphorylation and expression of Smad and P38. C2C12 cells were cultured with GSB and naringin in the test group, with noggin and dorsomorphin in the positive control group, and with dexamethasone and 077 in the negative control group. The protein was recognized as the phosphorylation of Smad and P38, and was used in Western blot analysis to detect phosphorylation change.

In Western blots (Figure 1), Smad phosphorylation was initiated when BMP-2 bound to BMPR-1A in C_2_C_12_ cells. In the presence of dorsomorphin (a BMPR signalling inhibitor) and Noggin (a BMP-2 binding protein), phosphorylation of Smad was decreased. Similar results were observed when cells were treated with GSB and naringin, indicating direct cell differentiation through blocking of BMPR signalling. However, negative controls treated with dexamethasone exhibited no decrease in the phosphorylation of Smad and P38 or the expression of proteins such as Smad and actin. The results suggested the need to further evaluate upstream signal transduction.

### 3.4. BMP-2 binding assay 

**Figure 2:**
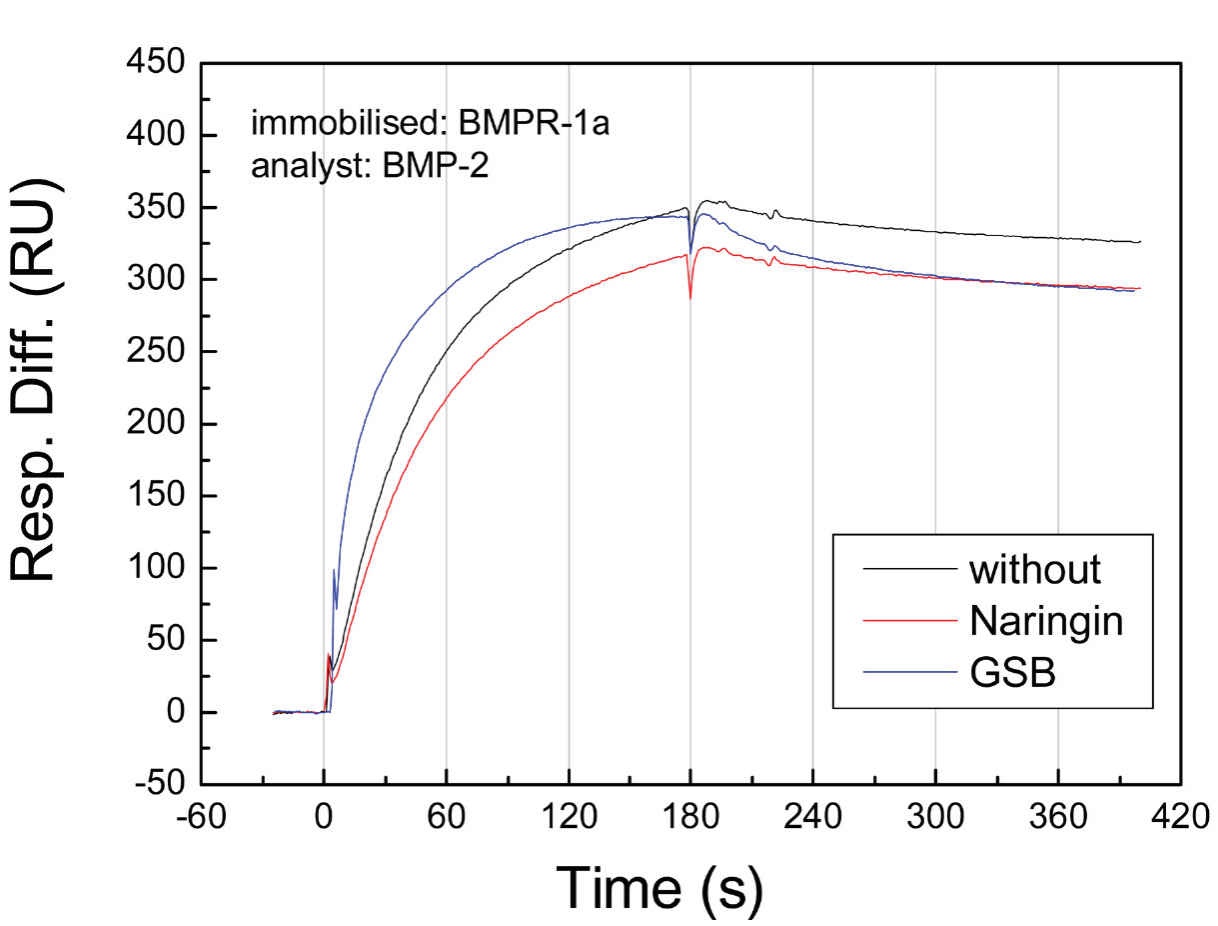
Binding curve of BMP-2 and BMPR in cells pretreated with naringin. BMPR-1A was immobilized on the SPR chip and maintained in a flow system at a flow rate of 10 μL/min. BMP-2 solution was injected into the flow system and passed through immobilized BMPR-1A. Binding was recorded in real-time based on the SPR response. Prior to BMP-2 injection, the test solutions were preinjected through the immobilized BMPR-1A. The black, red and blue curves represent BMP-2 binding alone and with preinjection of naringin and GSB solution, respectively.

BMP-2 has 2 natural binding receptors (BMPR-1A and BMPR-1B). BMPR-1A has a higher affinity for binding BMP-2 than that of BMPR-1B. The KD of BMPR-1A is slightly smaller than that of BMPR-1B. In SPR experiments (Figure 2 and Table), BMP-2 showed adequate binding to BMPR-1A, (KD = 1.56 nM). This result is consistent with the results of previous studies [30]. Interestingly, after pretreatment with GSB, the association curve of BMP-2 and BMPR-1A was slightly increased compared to the condition without treatment. Additionally, a more rapidly decreasing dissociation curve was observed. A general simulation of 1:1 binding showed a higher association rate constant (4.22 ×105 1/Ms with GSB treatment and 2.03 × 105 1/Ms without treatment) and a higher dissociation rate constant (5.67 × 10-4 1/s with GSB treatment and 3.17 × 10-4 1/s without treatment). The affinity for GSB was similar to that in the control group (KD=1.34 nM with GSB treatment and 1.56 nM without treatment). However, at a lower fitting level, the curve simulation showed a large chi-squared value of 106. Indeed, other molecular interactions may be induced through GSB treatment. Using a bivalent model, we again simulated the binding curve and observed adequate parameters with lower chi-squared values. The results showed that GSB may decrease the affinity of BMPR-1A/BMP-2 (KD = 2.71 nM with GSB treatment and 1.56 nM without treatment). This result suggested that some compounds in GSB interfere with the BMP-2/BMPR-1A interaction. To examine this hypothesis, naringin, a GSB marker compound, was evaluated using the same binding assay. The results showed that naringin also inhibited BMP-2 binding to BMPR-1A, but not as much as GSB, suggesting that naringin plays an important role in GSB-induced inhibition. In addition, other active compounds in GSB inhibit BMP-2 binding.

**Table:** Table. Binding parameters of BMP-2 binding to immobilized BMPR-1A pretreated with naringin. The data obtained from measurements using immobilized BMPR-1A were fit to a kinetic model (1:1 Langmuir binding) from which the K_D_ (bold) was calculated as kd/ka. All data represent the mean values of at least three repeated measurements using 6 different ligand concentrations.

BMP-2 binding parameters						
Pretreat with	ka (1/Ms)	kd (1/s)	Rmax (RU)	KA (1/M)	KD (M)	Chi^2^
Without	2.03e5	3.17e-4	363	6.41e8	1.56e-9	11.3
Naringin	1.77e5	3.82e-4	337	4.63e8	2.16e-9	10.7
GSB	4.22e5	5.67e-4	331	7.44e8	1.34e-9	106
GSB (Bivalent)	3.13e5	8.65e-4	375	3.62e8	2.71e-9	21.4

### 3.5. Preparation of the scaffold and tissue engineering tests

**Figure 3:**
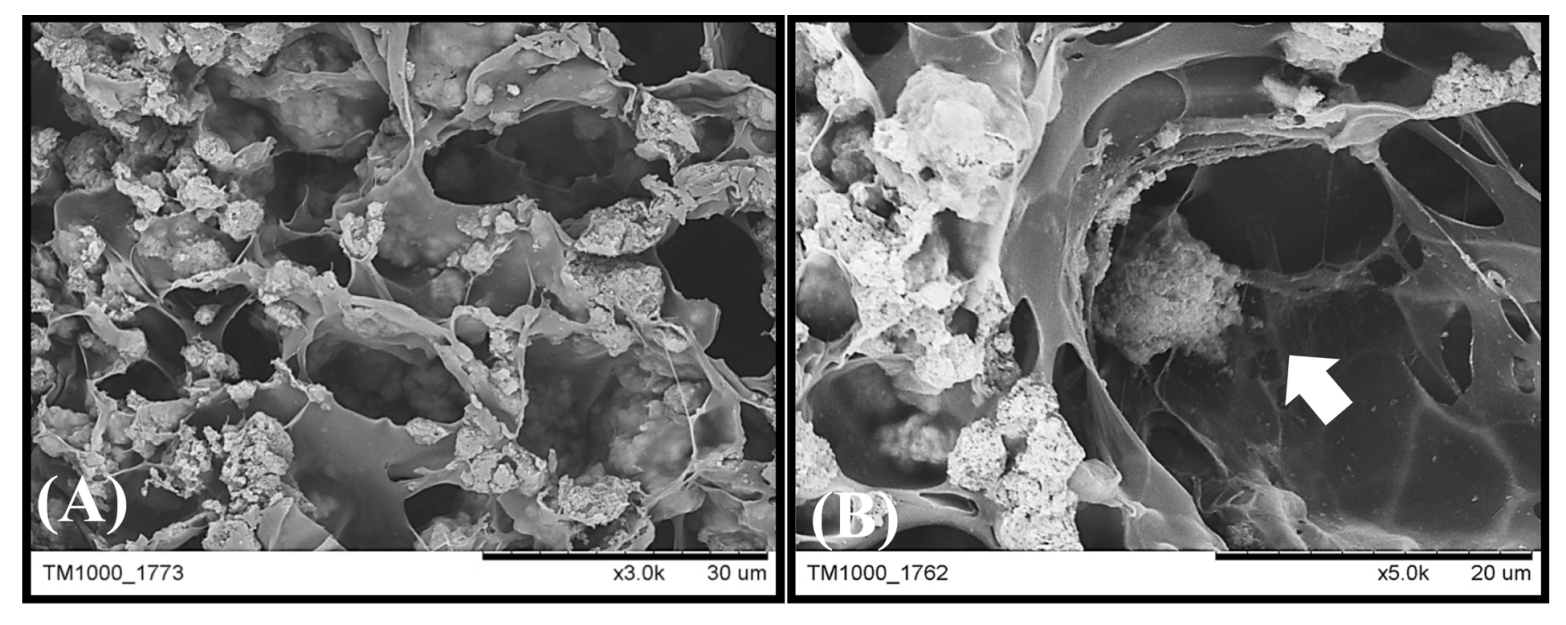
SEM micrographs of the *Nar* scaffold and cocultured mesenchymal stem cells. Rabbit bone marrow mesenchymal stem cells were cultured with *Nar* scaffolds in a dynamic bioreactor for 7 days. The scaffold (A) prior to culturing and (B) at 7 days after culturing was observed using SEM.

**Figure 4:**
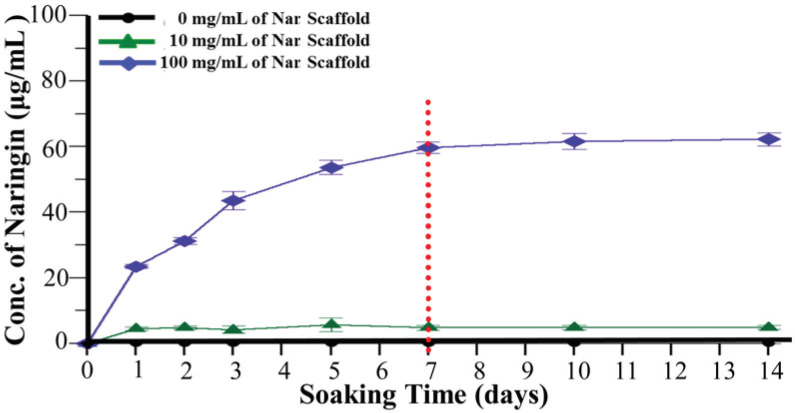
Time-release curves for the *Nar* scaffolds. Naringin was mixed with a GHG composite to prepare scaffolds with different concentrations of naringin (0, 10 and 100 mg/mL). The scaffolds were incubated in release test solution for 14 days. Thenaringin concentration in the solution was measured daily using HPLC. The black, green and blue colour curves represent 0, 10 and 100 mg/ mL *Nar* scaffolds, respectively. * means P < 0.05 compared with control and # means P < 0.05 compared with 10 mg/mL of Nar scaffold.

Naringin was added to the GHG scaffold preparation to form a novel naringin-containing scaffold (called *Nar* scaffold). In the SEM image analysis (Figure 3A), the average pore size of this *Nar* scaffold was 20 μm, similar to that of the GHG scaffold without naringin (called Cont. scaffold), indicating that the addition of naringin did not affect pore formation and Hap distribution in the Cont. scaffold. Additionally, rabbit BMSCs were cocultured with the *Nar* scaffold in a bioreactor for 1 week. The cells proliferated well in the pore of the scaffold, showing that this scaffold is bio-compatible (Figure 3B). In addition, a naringin release test was performed using different naringin-loaded scaffolds incubated in PBS for 14 days. Figure 4 shows that approximately 70 μg/mL of naringin can be released, and this amount was sufficient in induce BMSCs to differentiate into osteoblasts.

**Figure 5:**
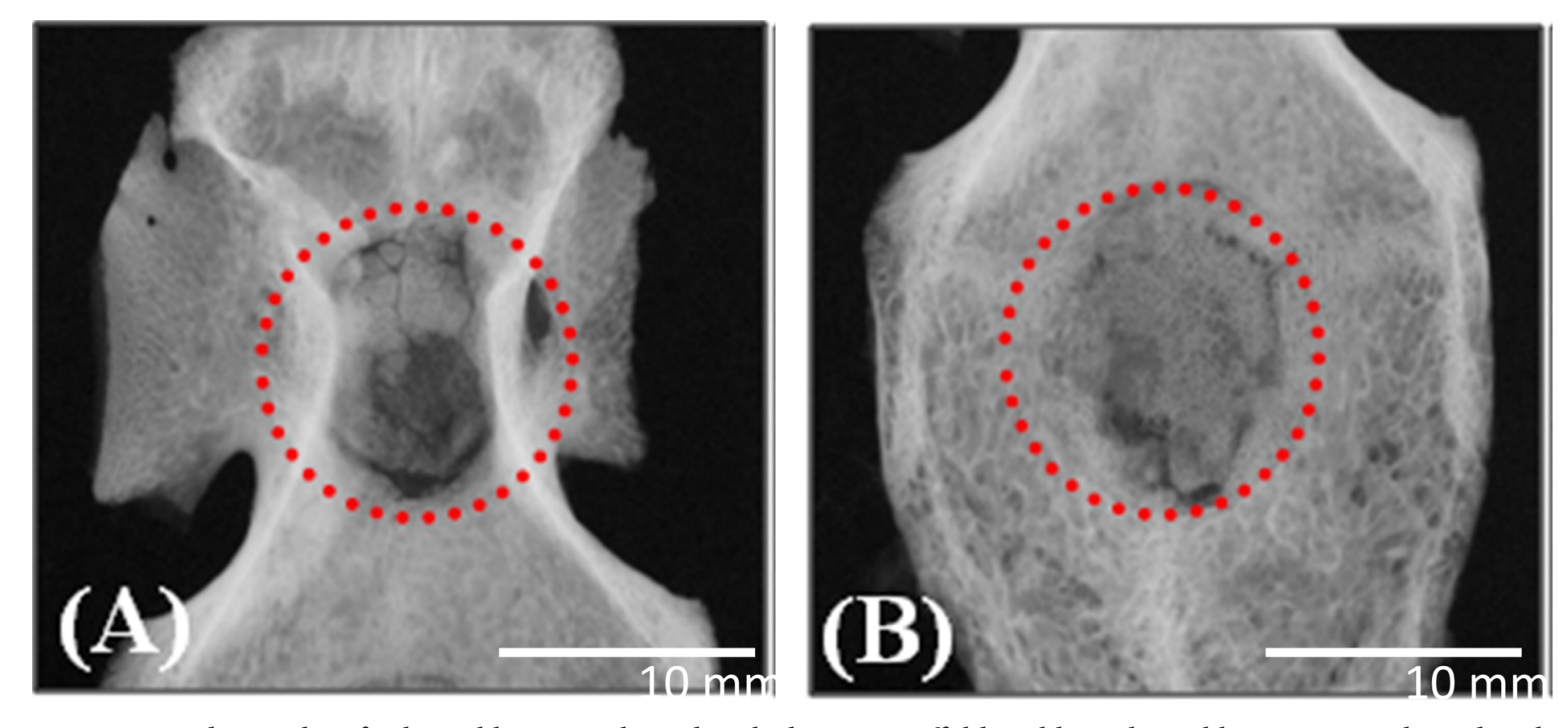
Radiographs of calvarial bone implanted with the *Nar* scaffold. Rabbit calvarial bone was implanted with (A) a *Cont* . scaffold and (B) a *Nar* scaffold for 4 weeks. The radiographs were obtained using mCT.

**Figure 6:**
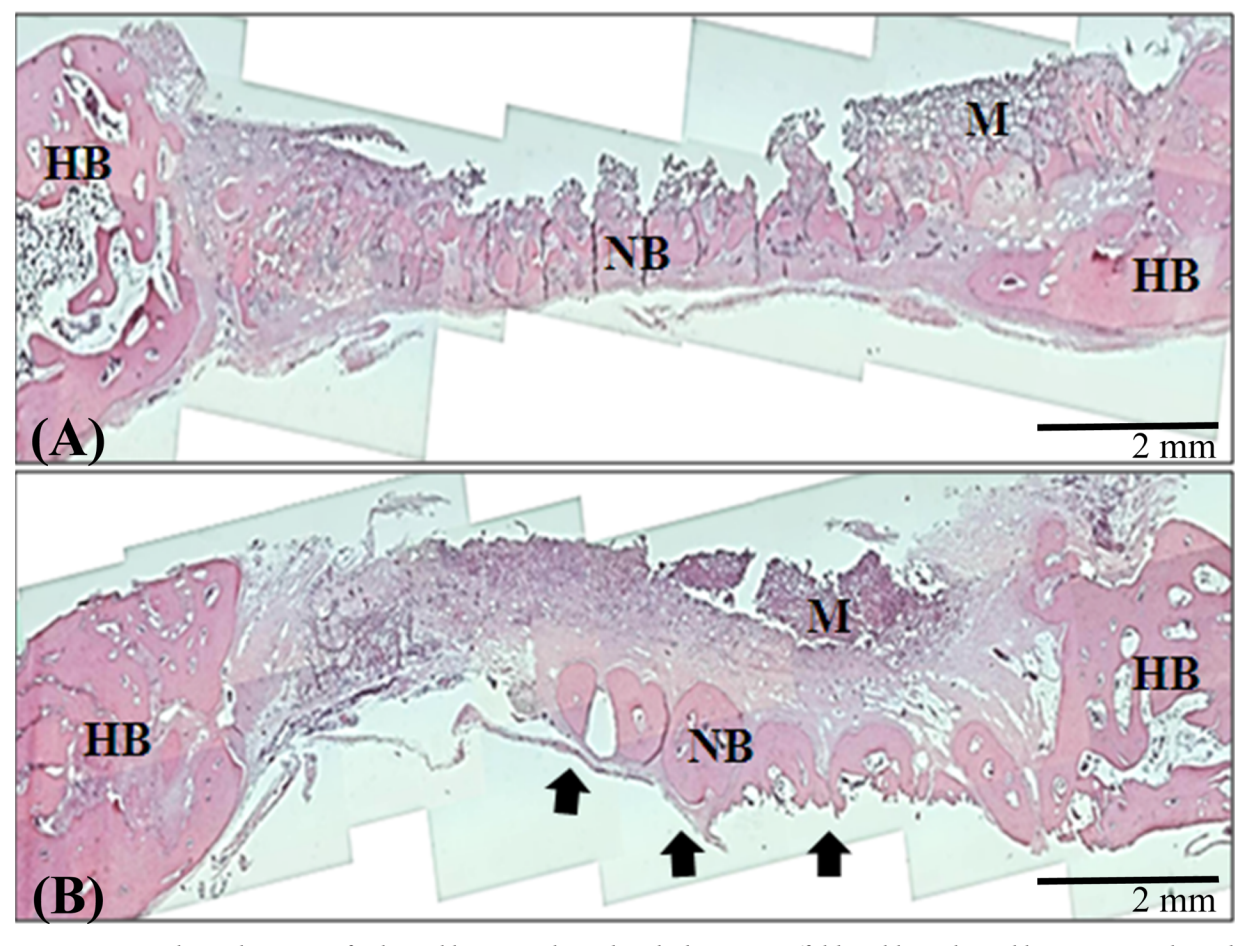
Histological images of calvarial bone implanted with the *Nar* scaffold. Rabbit calvarial bone was implanted with (A) a *Cont* . scaffold and (B) a *Nar* scaffold for 4 weeks. Histological images were obtained using an optical microscope.

Moreover, the *Nar* scaffold and *Cont.* scaffold were implanted into rabbit calvarial defects. After 4 weeks the rabbits were sacrificed, and bone regeneration in the defect was assessed. Figure 5A shows that the defect containing the control scaffold exhibited bone repair, and *Nar* scaffold improved osteogenesis (Figure 5B). In Figure 6, additional bone formation was observed in a histological examination. These results indicated that bone regeneration was not obvious by control scaffold implanted (Figure 6A), and naringin enhances bone regeneration in vivo (Figure 6B). 

The combined results of SPR and Western blotting experiments showed that naringin induced the osteogenic differentiation of BMSCs by blocking BMPR-1A signalling and directing BMP-2 to initiate BMPR-1B signalling. This mechanism is important for the ability of GSB to improve bone regeneration.

## 4. Discussion

For this study, we focus on the mechanism of Chinese herbal medicines GSB induced osteogenesis and used the marker compound naringin of GSB to incorporate on scaffold, and which proved the effective of osteogenesis. Naringin enhances BMP-2 induced-osteogenesis by the affinity of BMP-2 protein with BMPR-1A was reduced, due to naringin blocked BMPR-1A so that directed BMP-2 to initiate BMPR-1B signalling. By this result, we not only found out the binding relationship of receptor and molecular but also investigated the binding relationship affected protein bind with receptor.

In this study, we regulated the status of BMP-2 protein bind to BMPR through naringin, especially in naringin blocked BMPR-1A reacted with BMP-2 protein. BMPR-1A binding induces BMSC differentiation into fat cells. In contrast, BMPR-1B binding induces bone cell formation [30,35]. This is one of reason of naringin enhanced BMP-2 protein induced osteogenesis, and we proved the bone cell formation promotion by BMP-1B downstream related protein expression and animal experiment. In Lowery et al.’s study, they block BMPR2 to enhance BMP-2 protein function for enhance osteogenesis [36]. By the strategy, the competitive of BMP-2 protein and other protein to react with receptor would be enhanced. This concept is slightly similar to our research and we also inhibited the signalling by block one of coreceptor. We employed naringin as a receptor blocker to inhibit non-targeting downstream signalling that BMP-2 protein can improve effect on the targeting receptor BMPR-1A. 

The GHG scaffold incorporated with naringin as an implant to improve new bone formation. In previous study, GSB was incorporated with gelatin based scaffold and the required treatment time is 8 weeks [28]. We employed naringin as an osteogenic inducer of GHG scaffold and which could reduce treatment time from 8 weeks to 4 weeks. It was possible that naringin was a marker compound of GSB for osteogenesis so that it had better bone healing effect than GSB scaffold, other reasons maybe due to naringin had high released and stable delivered from scaffold to enhance bone healing.

This study investigated the mechanism of Chinese herbal medicine induced osteogenesis by binding property, especially in the active ingredient naringin of GSB and verified the effective on in vivo study. For other Chinese herbal medicine, we still unclean the mechanism and this also is the limitation, but the binding property can be developed a methodology to investigate the effective of medicine or molecular.

In conlusion, we investigated the affinity of the active ingredient naringin with receptor to explain the mechanism, and validated the active ingredient induced osteogenesis through animal experiments. In the present study, GSB and its marker compound (naringin) could inhibit the binding of BMP-2 and BMPR-1A to control cell differentiation. Furthermore, this binding blocked BMPR-1A signalling and enhanced BMPR-1B signalling, resulting in osteogenesis. Naringin was incorporated into a GHG composite to form a TCM-containing scaffold for bone tissue engineering. In animal experiments, the bone defect filled with a naringin scaffold exhibited better bone repair and new bone generation. According to these results, GSB and naringin could be good natural BMP regulators for bone tissue engineering.

## Acknowledgement/Disclaimers

This project was financially supported through a grant from the National Health Research Institutes, Taiwan (01A1-MEPP09-014) and Kaohsiung Medical University (KMU‐KI109004). The authors would like to thank the Taiwan Mouse Clinic for assistance with the X-ray imaging experiments.

## Conflict of interest

This study was presented at the Taiwan-Turkey Science Summit entitled “Translation of Cells, Nanomaterials and Signaling Molecules into Regenerative Medicine” between April 1 to 3, 2018.
